# Supplementary material for: Sexual health knowledge acquisition processes among very young adolescent girls in rural Malawi: Implications for sexual and reproductive health programs
Source: PLoS One. 2024 Feb 23;19(2):e0276416. doi: 10.1371/journal.pone.0276416 (PMC10889655; doi:10.1371/journal.pone.0276416)
Supplement: Attachment — Submitted filename: Response to Reviewers.docx [file pone.0276416.s004.docx]

| **Response to the Reviewers’ Comments** | | |
| --- | --- | --- |
| **Response to Reviewer 1’s comments** | | |
| **Comment** | **Description** | **Response** |
| 1. | It is imperative to indicate in the introduction and through-out the manuscript the importance of not only providing ‘appropriate’ information – but rather ‘accurate, consistent and age-appropriate information’. This would tie in with their findings around misinformation and partial-information which is not consistent. | Thank you for this important suggestion. We have added a paragraph in the introduction section on the importance of providing accurate, consistent, and age-appropriate information to the third paragraph in the introduction section. Please refer to lines 72 to 88 highlighted in yellow in the introduction section, lines 740 to 742 in the discussion section, and lines 764 to 766 in the Implications for SH programming section. |
| 2. | Although the intervention Girls Club/ Go Girl Club initiative is nested/embedded within the DREAMS initiative, a box or section describing it in detail would’ve helped put the intervention in context of the findings and discussion. A separate sub-section to describe or reference what the GOC was about, who delivered it, how often, where would be useful. Also, include the ages it targeted and how it recruited its participants would be useful information for the manuscript reader. [page 5 line 77-83] | Thank you for this important suggestion. We have added a thick description of the Go Girl Club initiative including a box describing the interventions for the age groups. Please refer to lines 112 to 125 in the introduction section highlighted in yellow. |
| 3. | Line 89. What was the justification of focusing on knowledge acquisition processes on VYA, was that what the intervention was about? Would not having older adolescents as a comparative group been a better approach maybe to shed more light on why it is important to understand knowledge acquisition processes in the VYA? | Thank you for this important observation. We have provided the justification for focusing on knowledge acquisition processes in this age group. Please refer to lines 75 to 88 highlighted in yellow. In this study, the aim was to assess the specific influence of club participation on sexual health knowledge acquisition among very young adolescent girls, as such we chose a comparison group of very young adolescent girls who did not participate in the clubs. The justification for using a comparison group of VYAs who did not participate in the GOCs has now been included. Please refer to the introduction section lines 132 to 135 highlighted in yellow. We also agree with the reviewer that having older adolescents as a comparison group would be another better approach. Therefore, we have now included a recommendation in the Limitations section that future studies exploring a similar question should consider having older adolescents as a comparison group. Please refer to line number 773-780 highlighted in yellow. |
| 4. | Line 93. The SEM is a good broad framework to use in general. However, for this study the Social Cognitive Learning Theory would’ve probably been better to show the interaction between the individual and the various layers of the SEM that the authors present but acknowledge how environment influences behavior (The Social Cognitive Learning Theory acknowledges the constant interaction that exists between the individual and his or her environment, both structural and social, to shape behavior). | Thank you for this very important suggestion. During the analysis we carefully considered both the social-cognitive theory and the social-ecological model, considering both their strengths and limitations. However, we chose to use the social-ecological model, its limitations notwithstanding.  We agree with the reviewer that the social cognitive theory may have enabled understanding of the cognitive processes that influence knowledge acquisition, however, the social cognitive theory has a limited emphasis on context. Furthermore, the social cognitive theory primarily focuses on individual cognitive processes and their interaction with the environment, through concepts like self-efficacy, observational learning, and self-regulation. However, as mentioned above, it places less emphasis on broader social, cultural, and environmental contextual factors that can significantly impact knowledge acquisition. Additionally, while the social cognitive theory acknowledges the role of social influences, such as modeling and social support, it may not fully capture the complexity of social interactions that occur in the process of knowledge acquisition. Adolescents' learning experiences are shaped by their interactions with peers, teachers, mentors, and other significant individuals.  Lastly, the social cognitive theory does not extensively address the influence of broader environmental contexts, such as community resources, school settings, and cultural norms. These factors play a crucial role in shaping learning opportunities and determining the availability of resources for knowledge acquisition. By not fully considering these environmental influences, the social cognitive theory may provide an incomplete understanding of the factors that contribute to or hinder knowledge acquisition among adolescents. |
| 5. | Line 105-108. Following from above comment, I found the SEM a bit too simplistic. Learning and knowledge acquisition is a complex process and involves cognition, self-efficacy, attitude etc. | Thank you for this important comment. Our view above notwithstanding, we have now described the limitations of the SEM in the Limitations sections. Further to this, we have included a recommendation for future studies to consider an integration of the two theories to have a more holistic exploration of the individual’s cognitive processes within the broader social and environmental contexts. Please refer to lines 779 to 780 in the Strengths and Limitations section. |
| 6. | Study setting line 114-121. It would be great if the authors indicate how the two districts were selected and add a few statistics to show the magnitude of HIV, HIV treatment gaps, OVC, early sexual initiation and school-drop-out rates. | Thank you for this important comment. We have indicated how the districts were selected. We have also added the statistics that were used by the DREAMs Initiative implementers as a justification for implementing the DREAMS initiative in these two districts. The statistics include HIV prevalence (13% and 16.3% among 15–49-year-olds, in Zomba and Machinga, respectively), high proportions of orphans and vulnerable children below the age of 18 (20.7% and 18.9%, in Machinga and Zomba respectively), high prevalence of early sexual initiation (20.1% and 24.1% in Machinga and Zomba respectively), high rates of childbearing during adolescence (26.1% and 31.6% among the 15-19-year-olds in Machinga and Zomba respectively), and high primary school drop-out rates among girls (17.3% and 12.6% in Machinga and Zomba respectively). Please refer to the Methods section, lines 97 to 103. |
| 7. | Line 130-145. It would be interesting to elaborate how these VYAs were approached for the study and how the assessment of ability to participate was conducted, considering that some expressed discomfort with the interviews and unavailability of participants. Also, in the discussion, the interviewers were indicated to have been a limitation – were they trained in conducting interviews with children, could the study have used other child friendly approaches to engage the children attention for longer and capture data rather than long interviews? Ranking, sorting and participatory methods, etc could’ve been employed. | We thank the reviewer for this important question. We have now elaborated on the approach that was used to recruit the participants (i.e., VYAs) and how their ability to participate in the study was assessed. Please refer to the sampling section, lines 176-177. And also lines 182 and 184. Regarding the limitation on interviewers: We have now clarified that all the interviews were conducted by the first author. We have now also explained that the age gap between the researcher (1^st^ author) and the respondents, and their age could have limited what they said in the interviews. The interviewer (first author) is a seasoned qualitative researcher who has extensive experience working and doing research with children. Moreover, we used the narrative inquiry approach, which is a participatory research method that uses a storytelling approach. This approach is deemed to be a friendly and appropriate approach for collecting qualitative data from children[1–3]. For instance, the story telling nature of a narrative inquiry helps in rapport building which allows children to tell stories in their own way and to focus on key issues that are important to them. Please refer to the Reflexivity section line number 241-252. Please also refer to the Strengths and Limitations section line number 754 to 763. |
| 8. | When was the study conducted and for long? | Thank you for this important question. The data collection for the study was conducted between 2018 and 2019 for a period of two months. We have included this information in the sampling and data collection section. Please refer to lines 184-185. |
| 9. | Line 157-159. The interviews were conducted in a private home in the community, where all the participants taken to this place? Why were the interviews not conducted within the children’s homes? I understand parental consent to participate in the GOC and interviews was obtained. I understand this was a sensitive study and may disclose VYA sexual behavior to the parents – but if parental consent was obtained, they would’ve known what the study was about and maybe VYA would’ve been comfortable doing interviews in their homes? Please clarify | Thank you for this important question. The decision to conduct the interviews at a private house was because we had concerns around privacy in their homes which would have possibly made the respondents to not speak freely and in a candidate manner when they knew that someone else could potentially overhear what they were saying. Also, we were concerned that at the children’s homes, siblings or friends might either want to listen in on the interview out of curiosity or keep interrupting the respondent for other reasons. As such, we decided that interviews will be conducted in a private place within the community. This was also a preferred approach by the Research Ethics Committees that approved this study. We have now included this information in the manuscript. Please refer to the sampling and data collection section, lines 204 to 209. |
| 10. | Results: Line 610 – I think the proportion of VYA not in school is worth noting in the discussion especially as the authors indicate schools were a source of info and VYA who were not in the GOC would’ve probably not received SH in schools. | Thank you for this important observation and suggestion. We have now included the proportion of VYAs who were not in school in the discussion section. Please refer to the discussion section lines number 708 to 709 highlighted in yellow. |
| 11. | Line 269-326. I found the findings quite repetitive and could’ve been easily combined. This is mostly a presentation issue and probably trying to frame the results around the SEM as no new themes emerged/were generated at different layers. | Thank you for this important comment. We agree that the data presented in lines 269-326 may seem repetitive. However, these data speak to three instances in which parents communicate to VYAs about SH issues. The first instance relates to how parents may provide random advice about SH issues to the VYAs. The second instance relates to communication and advice that was given to VYAs because the parent was suspicious that the VYA was engaging in a romantic relationship. The third instance shows advice that came from parents because the VYA girl was known to have started her menstruation. Please refer to line number 436 to 448, 449 to 469, and 409-417. |
| 12. | Line 293 – this sentence is not clear – please look and clarify. | Thank you for this important observation. We have revised the sentence. Please refer to line number 449-451. |
| 13. | Line 371 -379 – the quotes seem repeated – were there other quotes from the other VYA which can be drawn from? | Thank you for this important comment. We have revisited the quotes referred to and noted that they are two different quotes. The quote which was in line 371 (now 475 to 480) illustrates a discussion among VYAs which focused on describing the signs of a pregnant woman. The quote in line 379 (now 483-485) reflects a discussion among VYAs regarding the sources of information about HIV and how to prevent HIV. We have thus maintained the quotes as they are. |
| 14. | Line 441 – if you could please explain what the “school-based mother group clubs” are and how they are different from GOC, what they teach? How did they identify girls who would’ve started menstruation? | Thank you for this comment. The GOCs are clubs based in the communities sponsored by the DREAMS initiative as explained in lines 103 to 116 in the introduction section. On the other hand, the mother groups are volunteer women from the community who come to teach adolescent girls about SH and also encourage girls to remain in school. These are not part of the DREAMS initiative. We have added some references where more about mother groups have been explained. Please refer to line number 552 to 553, highlighted in yellow.  From the quotes in line lines 556 to 562, highlighted in yellow, the girls who started menstruation were identified in school classes where a teacher shared information with the girls above the availability of the mother groups who would be able to offer information and advice to the girls about menstruation. The teachers then encourage the girls who had started menstruation to speak to the mother groups. |
| 15. | Line 618 – I would probably add/rephrase the sentence so it is a more positive approach and ensure that knowledge and skills go beyond just protecting themselves but making informed decisions/choices regarding their sexual health. | Thank you for this important suggestion. We have revised this sentence as per the reviewer’s suggestion. Please refer to line number 710 to 712. |
| 16. | Line 636-644. For each limitation listed, it would be great to show why/how it was a limitation and show how the study went around he issues highlighted where possible. | Thank you for this important suggestion. In line 754 to 759 highlighted in green, we have described the limitations of the study highlighting four factors These factors related to the amount of time that the respondents had during the interview, and the limited time that the interviewer had to explore in-depth the issues and topics explored in this study. In line 760 to 763 highlighted in yellow, we have explained that the use of narrative inquiry which entailed that participants respond to questions in a story telling manner, helped to address some of these limitations. We have explained that the narrative inquiry approach helped in building rapport with the VYAs by allowing them to tell their stories in their own way and to focus on key issues that they felt were important to talk about. |
| **Response to Reviewer 2’s comments** | | |
| 17. | **Abstract and background** The explanation in the study setting section line 114-119 that gives the statement of the problem should be moved to the background section as it provides robust contextual information. “This study was carried out in Malawi's rural southern districts of Zomba and Machinga. Youth aged 10 to 24 make up one-third of the population in both districts (40). The districts were chosen because of their high HIV prevalence (13% and 16.3% among 15–49-year-olds, in Zomba and Machinga, respectively), HIV treatment gaps, high proportions of orphans and vulnerable children, high prevalence of early sexual initiation, high rates of childbearing during adolescence, and high school drop-out rates among girls (26).” | Thank you for this important suggestion. We have now moved this section to the introduction section. Please refer to the introduction section, fifth paragraph line number 97 to 103. |
| 18. | The rationale and the aim of the research need to be clearly articulated to connect the background with methodology. | Thank you for this important comment. The rationale and the aim have now been clearly articulated to connect background with methodology. Please refer to lines 126 to 132. |
| 19. | Can you dedicate a section in the methodology for a reflective account of your role in the DREAMS project, on the process of the interview, on the reaction of the VYAs to your questions, on what your expectations between the GOC and Non-GOC was, and how that changed during the data collection period and beyond? | Thank you for this important suggestion. We have added a reflexivity section in the methods section. Please refer to line number 228 to 252 highlighted in yellow. |
| 20. | **Result** In my opinion this is where your manuscript needs more work to clearly answer your research question and attend to the complexity of the phenomenon you are investigating: What knowledge did you examine -- e.g., overall awareness, knowledge on mode of transmission, perceived susceptibility, or knowledge on prevention? This needs to be clearly stated in the paper. | Thank you very much for this important comment. We have now added a description of the type of knowledge that we investigated in this study. Please refer to line 290 to 293 in the results section highlighted in yellow. |
| 21. | Though your primary research objective is to explore the knowledge acquisition process, the themes seem limited to the sources of SRH knowledge (parent, peers, school, clubs). I would expect a deeper examination of the knowledge acquisition processes (communication, learning, mentoring, attitudes, competence on verification of reliability of information etc) and how they are different between the SEM levels. Also, the multiplicity of the sources is not emphasized e.g., one VYA’s could have multiple sources of information and acquire knowledge through different processes. | Thank you for this important comment. We agree that the themes presented seem to be limited to the sources of SH knowledge for VYAs which were parents, peers, schools and clubs. To more clearly show the knowledge acquisition processes, we have revised the presentation of the themes by reorganizing into 3 categories in line with the three SEM levels: individual, interpersonal, and community level factors. Please refer to the results section from line number 364 to 366. |
| 22. | I see you have indicated in the limitation how interview with other stakeholders could have contributed to the model, however the use of SEM still should be justified by presenting the levels, how the various levels interact, what factors influenced VYAs knowledge acquisitions and behaviors? A summary table that shows the SEM levels and the themes, factors, knowledge acquisition processes, the sources of information at each level would be helpful. | Thank you for this important comment. We have now presented a summary table in the results section which depicts how the factors at the various levels of the SEM interact to influence the knowledge acquisition process among VYAs. Please refer to line number 354 to 355 |
| 23. | Line 631-633 “The study has several strengths. Firstly, the use of the SEM provided us with a framework for understanding how multiple and interacting factors affected the SH knowledge acquisition processes among the VYA girls.” I don’t see the multiple and interacting factors that affected the SH knowledge acquisition process? | Thank you for this important comment. In addressing the comment above, we have revised the themes and presented them in 3 categories according to the SEM. We trust that the revision we have done and Table 3 show more clearly the multiple and interacting factors at the different levels of the SEM and how these influence knowledge acquisition process among VYAs. Please refer to the results section from line number 365 to 600 and to Table 3. |
| 24. | Was it possible to conduct a comparison between the VYAs from the implementation sites and VYAs outside the implementation areas? Was there a difference in a source (apart from the clubs, did they communicate better with their parents and peers), was there a difference in the knowledge acquisition process, factors influencing the knowledge acquisition process, was the quality of knowledge different? Line 197 that themes were compared between the two groups. Could you present them? | Thank you for these important questions. We used a constant comparative analysis approach to compare the GOC participants and Non-GOC participants. We have added this explanation in the data analysis section line 264 to 272. The themes that we compared are listed in lines 264 to 267. In the results section line 582 to 643 we have presented the differences in relation to the sources of SH information and knowledge acquisition process, the accuracy or correctness of knowledge obtained, between club participants and Non-GOC participants. |
| 25. | What role did media and technology play if explored? Do the VYAs have access to media and technology, including mass media (radio and TV), social media etc..? | Thank you for this important question. We did not explore access to mass media and technology in this study. We have now acknowledged this as a limitation and provided a recommendation for future studies about the importance of exploring the role of media and technology as a source of SH for VYAs. Please refer to line number 768 to 771 |
| 26. | Minor comment 1. Your abstract is unstructured and a little bit difficult to follow. Can you provide a structured abstract and especially emphasis on the themes and the key findings from your analysis | Thank you for this important comment. For this manuscript, we used an unstructured abstract format because it is the journal requirement for PLOS ONE. |
| 27. | Can you provide the semi-structured interview guide as a supplementary material? | Thank you for this important request. We have provided the semi-structured guides used in the study as supplementary material. |
